# Cephalic Tetanus: A Case Report

**DOI:** 10.1155/2011/780209

**Published:** 2011-09-22

**Authors:** M. A. Alhaji, U. Abdulhafiz, C. I. Atuanya, F. L. Bukar

**Affiliations:** ^1^Department of Paediatrics, University of Maiduguri Teaching Hospital, PMB 1414, Maiduguri, Borno State, Nigeria; ^2^Amana Specialist Hospital, Tandari-Shokwari Ward, Maiduguri, Borno State, Nigeria; ^3^Department of Community Medicine, College of Medical Sciences, University of Maiduguri, PMB 1069, Maiduguri, Borno State, Nigeria

## Abstract

A case report of cephalic tetanus in a 2-year-old girl who was not immunized against tetanus following suppurative otitis media (SOM) is presented. This case is reported because of the rarity of cephalic tetanus associated with high mortality, to highlight the risk of cephalic tetanus as sequelae of SOM and the need for proper aural care and prompt treatment of SOM. Primary immunization of all eligible children as well as booster vaccination at appropriate time as an effective management strategy for tetanus is emphasized.

## 1. Introduction

Tetanus is a vaccine-preventable disease and remains an important cause of under five morbidity and mortality, especially in the developing countries [1–4]. Tetanus is considered as a failure of public health system. 

Tetanus is caused by an exotoxin, tetanospasmin, produced by *Clostridium tetani*. Diagnosis is mainly clinical, it may manifest as a generalised form, characterized by trismus, risus sardonicus, neck stiffness, dysphagia, and muscle rigidity and spasm, or localized form in which the spasms and rigidity are confined to anatomic area of the injury. Localized tetanus is rare in childhood [2, 5, 6]. Cephalic tetanus is a rare form of localized tetanus [7] occasionally occurring as a complication of SOM [8] or following craniofacial injuries [9]. In cephalic tetanus, paralysis of cranial nerves rather than spasms predominates but progression to generalised tetanus is not uncommon.

## 2. Case Presentation

A two-year-old unimmunized girl was seen in our clinic and admitted with a complaints of ten-day history of purulent left ear discharge, inability to open the mouth, and neck stiffness of five days duration associated with cough and fever of two days prior to presentation. There was no recent injury to the head and neck or uvulectomy. No previous history of SOM. She never received primary immunization with tetanus vaccine due to parental negligence.

Physical examination revealed an acutely ill-looking febrile child with trismus, neck stiffness, and frequent contact spasms of the facial muscles. There were no obvious spasms in other parts of the body. She was conscious, with left VII cranial nerve palsy, of lower motor neurone lesion. Otological examination revealed purulent discharge from the left ear with poor visualization of the ipsilateral tympanic membrane. She was dyspnoeic with wide spread coarse crepitations on chest auscultation. A diagnosis of cephalic tetanus with bronchopneumonia to rule out meningitis was made.

The microscopic examination of the otorrhoea yielded gram-negative bacilli. The biochemistry and microscopic profile of the cerebrospinal fluid was not suggestive of bacterial meningitis. Chest radiograph was not done. 

Treatment given consisted initially of dextrose saline and combination therapy with intravenous diazepam and intramuscular phenobarbitone, alternating 6 hourly to achieve sedation and control of spasms. Subsequently, the parenteral drugs were changed to oral after passing nasogastric tube for feeding and medications. 

Other medications given included oral penicillin, metronidazole, and intramuscular antitetanus serum. The ear was kept dry with regular aural toileting. She was nursed in a quiet room to avoid unnecessary stimuli (sound and light). 

The frequency of facial muscle spasms and trismus reduced over the next one week, and the nasogastric tube feeding was weaned-off.

She was discharged home on oral diazepam on request of her parents on the 9th day of admission because of pressing domestic reasons. The parents were counselled and the child was given primary immunization. She was seen one week at follow-up visit, with full recovery of the facial nerve palsy, no spasms, and minimal trismus. The otorrhoea had ceased.

## 3. Discussion

Cephalic tetanus is a rare form of localized tetanus defined as trismus plus paralysis of one or more cranial nerves [10] and accounts for 1–3 percent of the total number of reported cases of tetanus. Cephalic tetanus occasionally follows SOM [8], as with our patient, or craniofacial injuries [9]. About two-thirds of cephalic tetanus cases progress to generalised tetanus with bad prognosis [11]. In those who do not progress to generalized tetanus, the prognosis is good. However, our patient did not progress to generalized tetanus, with good outcome, probably, due to prompt intervention. This case is presented as there are not many previous reports of cephalic tetanus in childhood with good prognosis. 

Tetanus, as a disease of failed public health system due to inadequate immunization, has been cited by many studies [1–4]. The role of SOM as a portal of entry in postneonatal tetanus has been elucidated through many studies [1–4, 7]. 

Treatment consists of nursing care, aural toileting, sedation, muscle relaxation, and antibiotics, with good outcome. The patient recovered fully without progression to generalised tetanus and with minimal sequelae.

It is hereby recommended that early diagnosis and prompt treatment of SOM is important in preventing complications such as cephalic tetanus. The importance of both primary and booster immunizations cannot be overemphasized as an effective preventive management strategy in reducing tetanus in general.
